# Heterogeneity of the Relative Benefits of TICI 2c/3 over TICI 2b50/2b67: Are there Patients who are less Likely to Benefit?

**DOI:** 10.1007/s00062-021-01131-0

**Published:** 2022-01-06

**Authors:** Christoph C. Kurmann, Adnan Mujanovic, Eike I. Piechowiak, Tomas Dobrocky, Felix Zibold, Morin Beyeler, Jan Vynckier, David Seiffge, Thomas R. Meinel, Pasquale Mordasini, Marcel Arnold, Urs Fischer, Jan Gralla, Johannes Kaesmacher

**Affiliations:** 1 University Institute of Diagnostic and Interventional Neuroradiology, University Hospital Bern, Inselspital, University of Bern, Bern, Switzerland; 2 Department of Neurology, University Hospital Bern, Inselspital, University of Bern, Bern, Switzerland; 3 University Institute of Diagnostic and Interventional and Pediatric Radiology, University Hospital Bern, Inselspital, University of Bern, Bern, Switzerland

**Keywords:** Ischemic stroke, Mechanical thrombectomy, Cerebral vessel occlusion, Risk factors, Stent retriever

## Abstract

**Purpose:**

Incomplete reperfusion after mechanical thrombectomy (MT) is associated with a poor outcome. Rescue therapy would potentially benefit some patients with an expanded treatment in cerebral ischemia score (eTICI) 2b50/2b67 reperfusion but also harbors increased risks. The relative benefits of eTICI 2c/3 over eTICI 2b50/67 in clinically important subpopulations were analyzed.

**Methods:**

Retrospective analysis of our institutional database for all patients with occlusion of the intracranial internal carotid artery (ICA) or the M1/M2 segment undergoing MT and final reperfusion of ≥eTICI 2b50 (903 patients). The heterogeneity in subgroups of different time metrics, age, National Institutes of Health Stroke Scale (NIHSS), number of retrieval attempts, Alberta Stroke Programme Early CT Score (ASPECTS) and site of occlusion using interaction terms (p_i_) was analyzed.

**Results:**

The presence of eTICI 2c/3 was associated with better outcomes in most subgroups. Time metrics showed no interaction of eTICI 2c/3 over eTICI 2b50/2b67 and clinical outcomes (onset to reperfusion p_i_ = 0.77, puncture to reperfusion p_i_ = 0.65, onset to puncture p_i_ = 0.63). An eTICI 2c/3 had less consistent association with mRS ≤2 in older patients (>82 years, p_i_ = 0.038) and patients with either lower NIHSS (≤9) or very high NIHSS (>19, p_i_ = 0.01). Regarding occlusion sites, the beneficial effect of eTICI 2c/3 was absent for occlusions in the M2 segments (aOR 0.73, 95% confidence interval [CI] 0.33–1.59, p_i_ = 0.018).

**Conclusion:**

Beneficial effect of eTICI 2c/3 over eTICI 2b50/2b67 only decreased in older patients, M2-occlusions and patients with either low or very high NIHSS. Improving eTICI 2b50/2b67 to eTICI 2c/3 in those subgroups may be more often futile.

**Supplementary Information:**

The online version of this article (10.1007/s00062-021-01131-0) contains supplementary material, which is available to authorized users.

## Introduction

The association of better clinical outcomes and reduced long-term mortality with increased reperfusion quality of mechanical thrombectomy is well established [1–8]. A modified thrombolysis in cerebral infarction (mTICI) score of mTICI 2b and above (i.e. more than 50% reperfusion of the initially hypoperfused territory) has traditionally been considered successful [9]; however, mTICI 2b covers a wide range of reperfusion and the refined expanded treatment in cerebral ischemia (eTICI) scale subdivides it into eTICI 2b50 (50–66% reperfusion), eTICI 2b67 (67–89%) and eTICI 2c (near-complete, 90–99%). Accordingly, near complete (eTICI 2c) and complete (eTICI 3) reperfusion were associated with better clinical outcomes than eTICI 2b50/2b67 reperfusion [1, 10].

It remains less clear which patients with intraprocedural eTICI 2b50/2b67 reperfusion would benefit from rescue therapy and in which patients additional retrieval attempts may be futile/unnecessary and expose patients to unnecessary risks [11]. Recent work indicated that time metrics (particularly time from groin puncture to reperfusion) play a critical role in the effectiveness of complete reperfusion [12].

With the intention to explore a potential heterogeneity of the relative benefits of eTICI 2c/3 over eTICI 2b50/2b67, we analyzed outcome differences across different reperfusion grades in clinically important subpopulations.

## Material and Methods

### Study Population

**Fig. 1:**
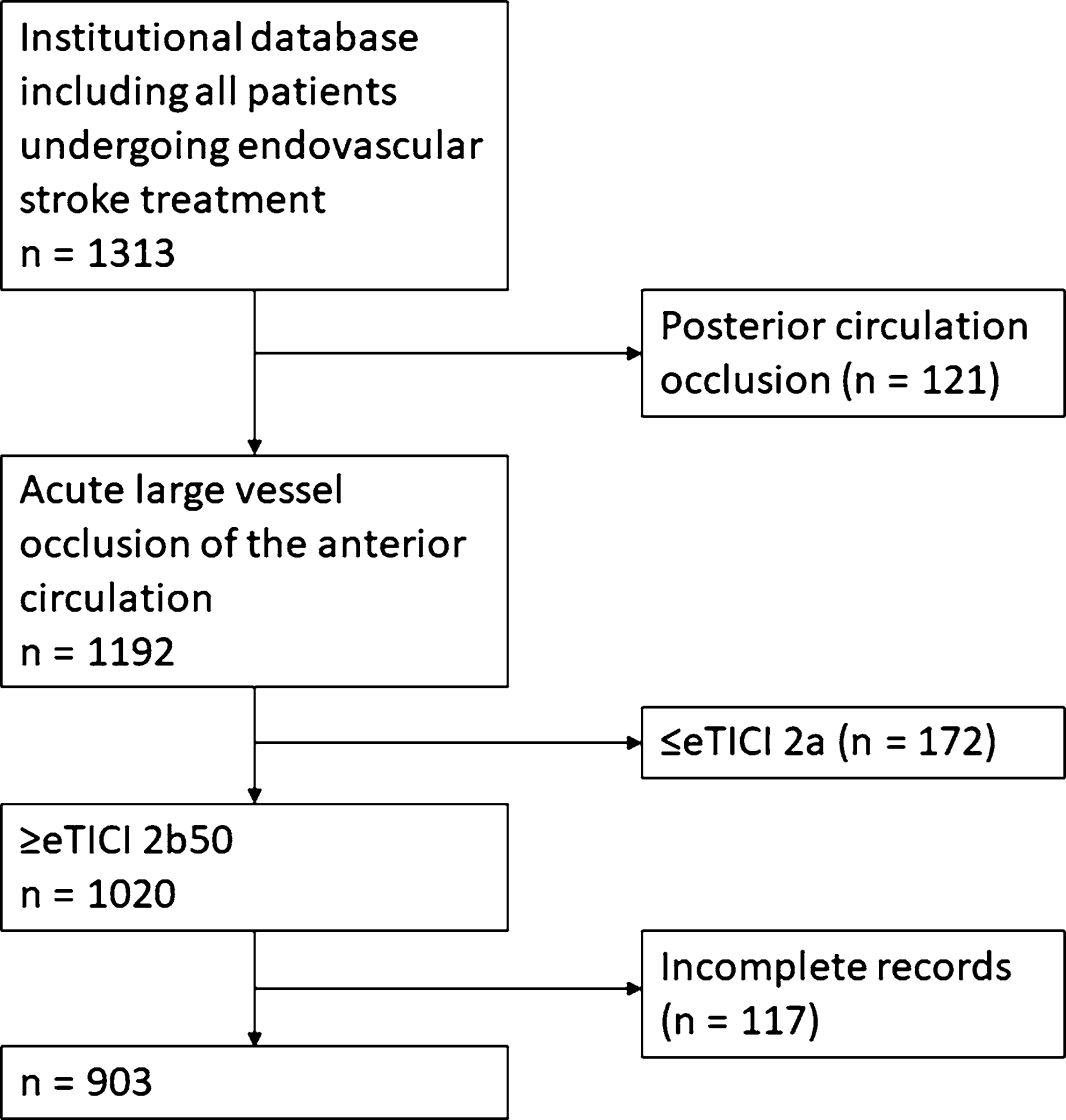
Flowchart showing the patient selection procedure

We performed a retrospective analysis of our institutional database including all patients with occlusion of the intracranial internal carotid artery (ICA) or the M1/M2 segment, undergoing endovascular stroke treatment and final reperfusion degree of ≥50% (≥eTICI 2b50) between 1^st^ January 2010 and 31^st^ December 2018. Patients with incomplete/insufficient angiographic documentation of preinterventional and postinterventional digital subtraction angiography (DSA) runs as well as incomplete clinical history were excluded (Fig. 1).

### Image Analysis

The thrombolysis in cerebral infarction (TICI) scale visually estimates the grade of reperfusion of the initially hypoperfused territory (target downstream territory [TDT]), represented as restored antegrade capillary blush on DSA [13]. To assess the grade of reperfusion, we used the eTICI scale which makes the following distinctions: eTICI 0 (no reperfusion), eTICI 1 (thrombus reduction without any reperfusion of distal arteries), eTICI 2a (<50% reperfusion), eTICI 2b50 (50–66% reperfusion), eTICI 2b67 (67–89% reperfusion), eTICI 2c (near complete reperfusion, 90–99%), and eTICI 3 (100%) [1].

In the first step, three neuroradiologists independently evaluated the eTICI scores for 142 randomly selected patients based on preprocedural and postprocedural DSA runs, with both an anterior-posterior and a lateral projection. They were blinded to other clinical, imaging, and outcome data. Since good interrater reliability was achieved, the remaining cases (*n* = 761/903) were evaluated independently by core laboratory judication in the same fashion.

Collateral status was graded by two neuroradiologists based on the preinterventional DSA runs, blinded to all clinical and outcome information. Collaterals were graded according to the American Society of Interventional and Therapeutic Neuroradiology/Society of Interventional Radiology (ASITN/SIR) scale: 0—No collaterals visible to ischemic site, 1—Slow collaterals to the periphery of ischemic site, with persistence of some of the defect, 2—Rapid collaterals to the periphery of ischemic site, with persistence of some of the defect, and to only a portion of the ischemic territory, 3—Collaterals with slow but complete angiographic blood flow of the ischemic bed by the late venous phase, 4—Complete and rapid collateral blood flow to the vascular bed in the entire ischemic territory by retrograde perfusion [14].

### Statistical Analysis

Baseline and outcome parameters between the groups were compared using the Kruskal-Wallis test for continuous variables or the Fisher’s exact test for categorical variables. Data are displayed as median (interquartile range [IQR]) and *n* (%) if not otherwise specified.

Interrater reliability was assessed using Krippendorff’s alpha for multiple raters. Krippendorff’s alpha is a coefficient representing the interrater reliability for any number of raters and any kind of variables. Values were interpreted as follows: 0 = poor; 0.01–0.20 = slight; 0.21–0.40 = fair; 0.41–0.60 = moderate; 0.61–0.80 = substantial and 0.81–1.0 = almost perfect agreement.

The distribution of clinical outcomes (mRS at 90 days after the intervention) for each eTICI was depicted graphically. Furthermore, binary logistic regression modeling was performed to assess the association of eTICI and clinical outcome (dichotomized into mRS ≤2 = good outcome versus mRS >2 = poor outcome [15]) and to account for potential prespecified confounders based on the literature (age, sex, NIHSS on admission, number of retrieval attempts, site of intracranial occlusion, general anesthesia, time from onset to groin puncture and ASPECTS).

The cases were then grouped into eTICI 2b50/2b67 and eTICI 2c/3. A multiplicative interaction term (eTICI*variable) was used to test for interaction with the following variables: continuous time metrics (onset to reperfusion time, puncture to reperfusion time and onset to puncture time), age on admission, NIHSS on admission, number of retrieval attempts, ASPECTS, ASITN/SIR DSA collateral score and site of occlusion.

To assess heterogeneity, subgroups were defined based on their quartiles for time metrics, age, and NIHSS; for ASPECTS as 0–4, 5–7, and 8–10 [16]; for collaterals ASITN/SIR 0/1, 2, and 3/4 (due to the small number of cases with ASITN/SIR 0 and 4, respectively); and for the number of retrieval attempts as 1, 2, and ≥3. Finally, marginal effects were plotted for time metrics and age as well as point estimates for ASPECTS.

All statistical analyses were conducted using R (Version 4.0.2, R Foundation for Statistical Computing, Vienna, Austria) [17]. A two-tailed *p*-value of <0.05 was considered statistically significant.

## Results

### Population

A total of 903 patients with occlusion of the intracranial ICA or M1/M2 segments (median age 75 years, IQR 63–82 years, 51% female) were included. Baseline characteristics are shown in Table 1. Group sizes were almost balanced, only the eTICI 2b50 group was smaller. With higher degrees of reperfusion, there was a trend towards shorter time from symptom onset to groin puncture (eTICI 2b50 median 250.5 min (IQR 175.5–383.5 min), eTICI 2b67 median 238.5 min (IQR 170–355.25 min), eTICI 2c median 233.5 min (IQR 168–358.75 min), eTICI 3 median 213 min (IQR 154–313 min), overall *p* = 0.03) and a lower number of retrieval attempts (overall *p* < 0.001). Patients with NIHSS ≤9 presented with better collaterals (median ASITN/SIR 3, IQR 2–4) than patients with NIHSS 10–19 (median ASITN/SIR 1, IQR 1–2, *p* < 0.001) and consequently, patients with NIHSS ≥20 had worse collaterals (median ASITN/SIR 1, IQR 1–2) than patients with NIHSS 10–19 (*p* < 0.001) (Suppl. Table I). Except for a higher proportion of current/history of smoking in the eTICI 2b67 population, the groups were well balanced regarding risk factors and stroke etiology.

**Table 1:** Patient characteristics overall and stratified by eTICI scores

	All (*N* = 903)	eTICI 2b50 (*n* = 118)	eTICI 2b67 (*n* = 272)	eTICI 2c (*n* = 258)	eTICI 3 (*n* = 255)	*p*
*Age (years)*	75 (63–82)	74 (60.25–83)	75 (63.75–81)	73 (62–82)	76 (64–83.5)	0.37
*Sex (female)*	464 (51.4)	70 (59.3)	131 (48.2)	136 (52.7)	127 (49.8)	0.21
*Admission NIHSS*	15 (9–19)	15 (10–19)	15 (9.75–20)	15 (9–20)	15 (9.5–19)	0.78
*Admission imaging modality (MRI)*	481 (53.3)	78 (66.1)	134 (49.3)	128 (49.6)	141 (55.3)	0.01*
*Admission ASPECTS*	8 (6–9)	7 (6–9)	8 (7–9)	8 (6–9)	8 (7–9)	0.02*
*Onset to puncture (min)*	232 (165.5–350)	250.5 (175.5–383.5)	238.5 (170–355.25)	233.5 (168–358.75)	213 (154–313)	0.03*
*Number of retrieval attempts*						<0.001***
1	527 (58.4)	46 (39.0)	140 (51.5)	165 (64.0)	176 (69.0)	
2	208 (23)	36 (30.5)	78 (28.7)	48 (18.6)	46 (18.0)	
≥3	168 (18.6)	36 (30.5)	54 (19.9)	45 (17.4)	33 (12.9)	
*Site of intracranial occlusion*						0.33
Intracranial ICA	36 (4)	1 (0.8)	17 (6.2)	11 (4.3)	7 (2.7)	
T or L occlusion of ICA	210 (23.3)	23 (19.5)	57 (21.0)	67 (26.0)	63 (24.7)	
M1	514 (56.9)	65 (55.1)	162 (59.6)	145 (56.2)	142 (55.7)	
M2	143 (15.8)	29 (24.6)	36 (13.2)	35 (13.6)	43 (16.9)	
*ASITN/SIR DSA collateral score*						0.156
0	61 (6.8)	8 (6.8)	22 (8.1)	14 (5.4)	17 (6.7)	
1	355 (39.3)	41 (34.7)	99 (36.4)	99 (38.4)	116 (45.5)	
2	250 (27.7)	34 (28.8)	78 (28.7)	83 (32.2)	55 (21.6)	
3	221 (24.5)	33 (28.0)	69 (25.4)	54 (20.9)	65 (25.5)	
4	16 (1.8)	2 (1.7)	4 (1.5)	8 (3.1)	2 (0.8)	
*Intubation narcosis*	640 (70.9)	82 (69.5)	193 (71.0)	188 (72.9)	177 (69.4)	0.83
*3‑month mRS ≤2*	434 (48.1)	39 (33.1)	126 (46.3)	123 (47.7)	146 (57.3)	<0.001***
*Risk factors*						
Diabetes	148 (16.4)	19 (16.1)	42 (15.5)	42 (16.3)	45 (17.6)	0.93
Arterial hypertension	638 (70.7)	80 (67.8)	197 (72.7)	176 (68.2)	185 (72.5)	0.53
Dyslipidemia	513 (56.9)	64 (54.7)	157 (57.7)	137 (53.1)	155 (60.8)	0.34
Smoking history	209 (23.3)	27 (22.9)	79 (29.4)	53 (20.5)	50 (19.8)	0.04*
Previous stroke	114 (12.6)	18 (15.3)	39 (14.3)	26 (10.1)	31 (12.2)	0.39
Coronary heart disease	184 (20.5)	20 (16.9)	53 (19.6)	47 (18.3)	64 (25.2)	0.15
*Stroke cause*						0.14
Atherosclerosis	97 (10.8)	11 (9.3)	36 (13.3)	30 (11.6)	20 (7.9)	
Cardioembolic	407 (45.2)	42 (35.6)	126 (46.5)	111 (43.0)	128 (50.4)	
Other, determined etiology	56 (6.2)	11 (9.3)	14 (5.2)	17 (6.6)	14 (5.5)	
Undetermined etiology	341 (37.8)	54 (45.8)	95 (35.1)	100 (38.8)	92 (36.2)	
*IVT bridging*	366 (40.5)	43 (36.4)	106 (39.0)	102 (39.5)	115 (45.1)	0.33

Data are displayed as median (IQR) and *n* (%) if not otherwise specified

*NIHSS* National Institutes of Health Stroke Scale, *ASPECTS* Alberta Stroke Programme Early CT score, *ICA* internal carotid artery, *mRS* modified Rankin Scale, *IVT* intravenous thrombolysis, *MRI* magnetic resonance imaging, *ASITN/SIR* American Society of Interventional and Therapeutic Neuroradiology/Society of Interventional Radiology, *DSA* digital subtraction angiography, *eTICI* expanded treatment in cerebral ischemia

*p < 0.05, ***p < 0.001

### Degree of Reperfusion and Clinical Outcome

**Fig. 2:**
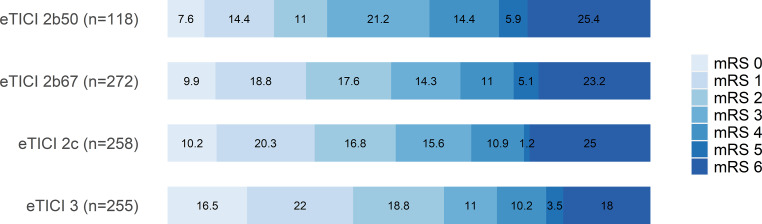
Percentage of patients with respective clinical outcome (*mRS*) 90 days after ischemic stroke, stratified by final eTICI scores

Higher degrees of eTICI were associated with mRS 90-day ≤2 (eTICI 2b50 as Reference, Table 2): eTICI 3 aOR 3.21 (95% CI 1.86–5.61), eTICI 2c aOR 1.91 (1.12–3.31), and eTICI 2b67 aOR 1.94 (1.14–3.33) (Fig. 2). Similarly, the proportion of patients with severe disability or death (mRS 5–6) decreased with higher eTICI scores, as did the proportion of patients with an intermediate level of disability (mRS 3–4).

**Table 2:** Multivariable logistic regression model with adjusted odds ratios (*aOR*) for good clinical outcome (mRS90-day ≤2)

Variable	aOR	95% CI	*p*
*Age on admission (per year)*	0.93	0.92–0.95	<0.001***
*Male gender*	1.04	0.78–1.46	0.80
*NIHSS on admission (per point)*	0.92	0.9–0.95	<0.001***
*ASPECTS*	1.33	1.22–1.46	<0.001***
*Site of occlusion*			
Intracranial ICA	Reference		
T or L occlusion of ICA	1.09	0.46–2.43	0.83
M1	1.45	0.63–3.13	0.36
M2	1.06	0.42–2.42	0.90
*Intubation narcosis (yes)*	0.87	0.6–1.23	0.44
*Onset to puncture (per min)*	0.99	0.998–0.999	0.02*
*Number of maneuvers*			
1	Reference		
2	0.64	0.45–0.96	0.02*
≥3	0.65	0.43–0.99	0.049*
*eTICI score*			
eTICI 2b50	Reference		
eTICI 2b67	1.94	1.14–3.33	0.02*
eTICI 2c	1.91	1.12–3.31	0.02*
eTICI 3	3.21	1.86–5.61	<0.001***

*NIHSS* National Institutes of Health Stroke Scale, *ASPECTS* Alberta Stroke Programme Early CT score, *ICA* internal carotid artery, *eTICI* expanded treatment in cerebral ischemia

*p < 0.05, ***p < 0.001

Interrater agreement among the 3 neuroradiologists for eTICI rating of 142 patients reached almost perfect agreement (Krippendorff’s alpha = 0.87, 95% CI 0.83–0.91).

### Heterogeneity of the Association of TICI2c/3 and good Clinical Outcomes with Strata of Time Metrics

**Fig. 3:**
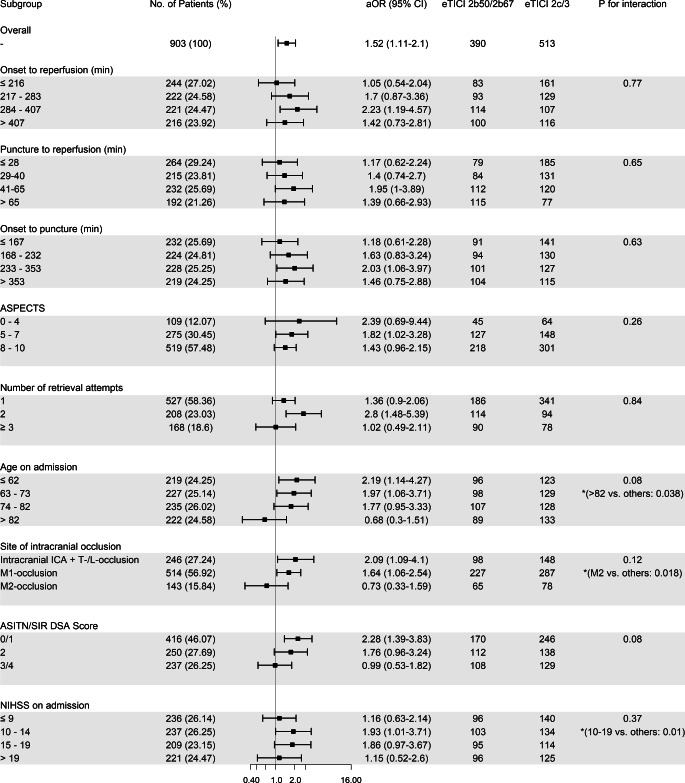
Adjusted odds ratios (*aOR*) for eTICI 2c/3 reperfusion for good clinical outcome (mRS-90 day ≤2) according to various time course metrics and baseline characteristics. Adjusted odds ratios, 95% CI, and *p*-values for interaction are shown. *Asterisk* Exploratory *p*-values show interaction for age >82 years vs. ≤82 years, M2 occlusions vs. ICA/T/L/M1 occlusions, and NIHSS 10–19 vs. NIHSS ≤9/>19 pooled. *CI* confidence interval

Time metrics as continuous variables showed no interaction with dichotomized eTICI scores (eTICI 2b50/2b67 vs. eTICI 2c/3; *p* for interaction: time from onset to reperfusion *p* = 0.77, time from groin puncture to reperfusion *p* = 0.65, time from onset to groin puncture *p* = 0.63). The adjusted odds ratios (aOR) for mRS 90-day ≤2 stratified by dichotomized eTICI scores slightly changed between different quartiles but showed no clear trend (Fig. 3).

**Fig. 4:**
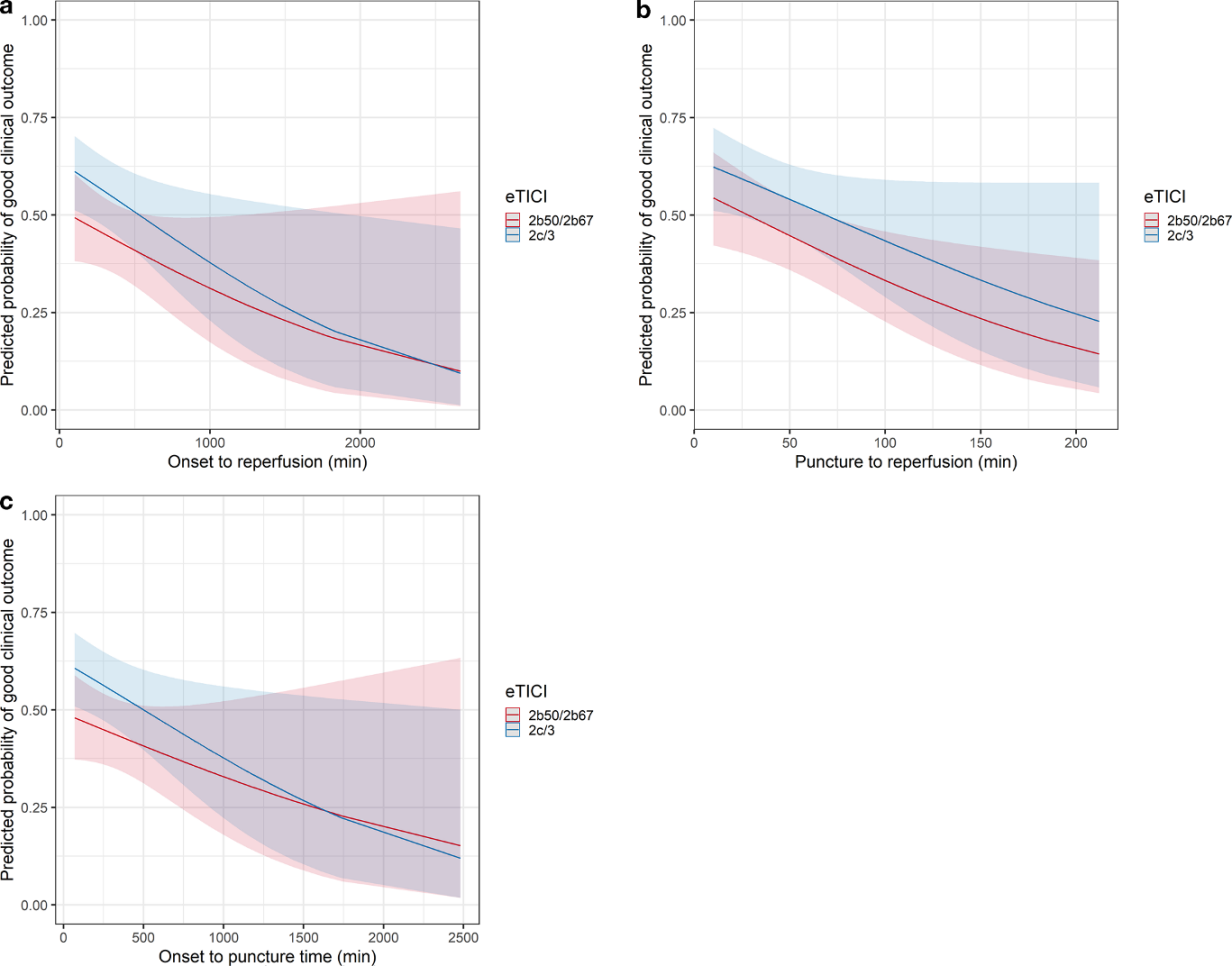
Predicted probabilities of good clinical outcome (mRS-90d ≤2) **a** on onset to reperfusion time, **b** on groin puncture to reperfusion time, **c** on onset to groin puncture time, stratified by eTICI, using multivariable logistic regression adjusted to age, sex, NIHSS on admission, number of retrieval attempts, site of intracranial occlusion, intubation narcosis, ASPECTS, and an multiplicative interaction term for each corresponding continuous time metric. The regression lines and 95% CI are shown

The change of probability of a good clinical outcome (mRS-90d ≥2) over time courses stratified by dichotomized eTICI scores are shown in Fig. 4 and show stable superiority of eTICI 2c/3 over eTICI 2b50/2b67.

We also observed a decreasing proportion of patients with eTICI 2c/3 reperfusion with increasing time metrics, probably most striking in the subgroups of the puncture to reperfusion time: 185/264 (70.1%) patients achieved eTICI 2c/3 in the group with ≤28 min, 131/215 (60.9%) patients in the group with 29–40 min, 120/232 (51.7%) patients in the group with 41–65 min and only 77/192 (40.1%) patients in the group with >65 min.

### Heterogeneity of the Association of TICI2c/3 and good Clinical Outcomes with Strata of Other Baseline Characteristics

Older age groups showed a trend towards decreasing beneficial effect of eTICI 2c/3 over eTICI 2b50/2b67 (*p* for interaction = 0.08), adjusted odds ratios for each quartile were as follows (Fig. 3): ≤62 years aOR 2.19 (95% CI 1.14–4.27), 63–73 years aOR 1.97 (95% CI 1.06–3.71), 74–82 years aOR 1.77 (95% CI 0.95–3.33), >82 aOR 0.68 (95% CI 0.3–1.51). There was a statistically significant interaction of effect when comparing the last quartile to the three others pooled (*p* = 0.038). These findings did not change substantially in a subanalysis including only patients with prestroke mRS ≤2 (*n* = 802, *p* for interaction = 0.09): ≤62 years aOR 2.06 (95% CI 1–4.23), 63–73 years aOR 2.24 (95% CI 1.11–4.59), 74–82 years aOR 1.95 (95% CI 1.01–3.84), and >82 years aOR 0.69 (95% CI 0.29–1.65).

There was no trend in change of beneficial effect of eTICI 2c/3 in quartiles of the number of retrieval attempts (*p* for interaction = 0.84) and no statistically significant interaction for NIHSS on admission when quartiles were handled as independent groups (*p* for interaction = 0.37). In patients with low NIHSS (≤9, aOR 1.16, 95% CI 0.63–2.14) and patients with high NIHSS (>19, aOR 1.15, 95% CI 0.52–2.6) however, eTICI 2c/3 had less impact on good clinical outcome as opposed to the middle groups (NIHSS 10–19, aOR 1.91, 95% CI 1.31–2.82, *p* for interaction = 0.01). In a further subgroup analysis of the low NIHSS group, patients with NIHSS ≤5 benefited less from eTICI 2c/3 (*n* = 103, aOR 0.34, 95% CI 0.11–1.03) than patients with NIHSS 6–9 (*n* = 133, aOR 2.08, 95% CI 0.9–4.93).

We found an increasing beneficial effect of eTICI 2c/3 with decreasing collateral scores (ASITN/SIR 3/4 aOR 0.99, 95% CI 0.53–1.82, ASITN/SIR 2 aOR 1.76, 95% CI 0.96–3.24 and ASITN/SIR 0/1 aOR 2.28, 95% CI 1.39–3.83, *p* for interaction 0.08).

There was no heterogeneity regarding the association of eTICI 2c/3 with good clinical outcomes across ASPECTS (Fig. 3). The point estimate of eTICI 2c/3 vs. eTICI 2b50/2b67 regarding good clinical outcomes was aOR 2.39 (95% CI 0.69–9.44) in ASPECTS 0–4 compared to a point estimate of aOR 1.82 (95% CI 1.02–3.28) and aOR 1.43 (95% CI 0.96–2.15) in ASPECTS 5–7 and 8–10, respectively (*p* for interaction = 0.26).

**Fig. 5:**
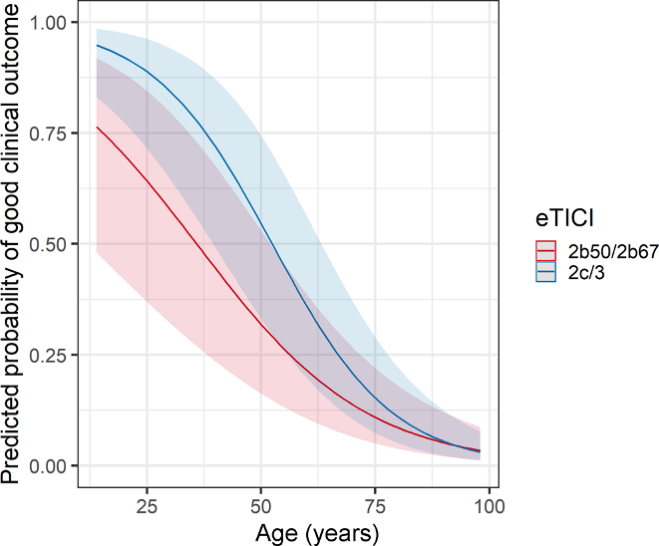
Predicted probabilities of good clinical outcome (mRS-90d ≤2) on age on admission, stratified by eTICI, using multivariable logistic regression adjusted to age, sex, NIHSS on admission, number of retrieval attempts, site of intracranial occlusion, intubation narcosis, ASPECTS, time from onset to puncture and a multiplicative interaction term for age. The regression lines and 95% CI are shown

**Fig. 6:**
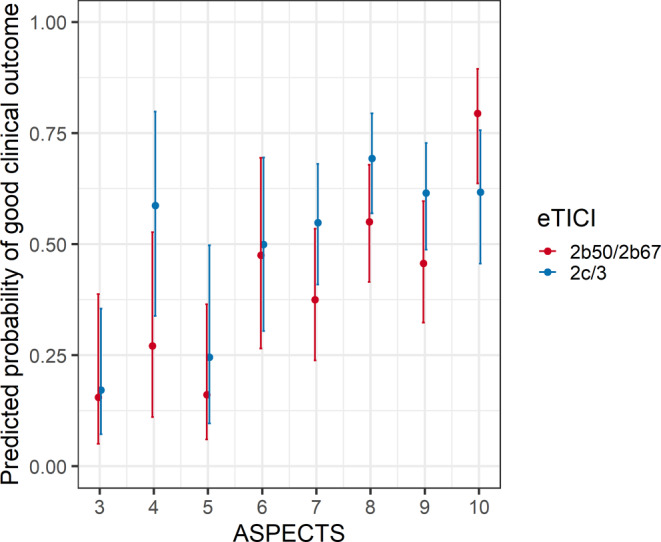
Predicted probabilities of good clinical outcome (mRS-90d ≤2) on ASPECTS, stratified by eTICI, using multivariable logistic regression adjusted to age, sex, NIHSS on admission, number of retrieval attempts, site of intracranial occlusion, intubation narcosis, ASPECTS, time from onset to puncture and a multiplicative interaction term for ASPECTS. The point estimates and 95% CI are shown

The change of probability of a good clinical outcome (mRS ≤2) over age (Fig. 5) and ASPECTS (Fig. 6) stratified by dichotomized eTICI scores again showed stable superiority of eTICI 2c/3 over eTICI 2b50/2b67 with the exception of older patients and patients with ASPECTS 10.

We found beneficial effects of eTICI 2c/3 in occlusions of the intracranial ICA/T or L occlusions (aOR 2.09, 95% CI 1.09–4.1) and of the M1 segments (aOR 1.64, 95% CI 1.06–2.54); however, this association was absent in patients with M2 occlusions (aOR 0.73, 95% CI 0.33–1.59, *p* for interaction vs. other occlusion sites pooled = 0.018).

## Discussion

This study has the following main findings: 1) better eTICI scores were associated with better clinical outcomes across several subgroups, including a large range of early ischemic changes and various time courses, and 2) a decreasing beneficial effect of eTICI 2c/3 over eTICI 2b50/2b67 was only found in older patient groups, more distal occlusions and patients with either low or very high NIHSS.

### Effect of near Complete/Complete Reperfusion in Subgroups

In line with previous work [1, 10], we found better outcomes for patients with ≥eTICI 2b67 reperfusion, when compared to patients with eTICI 2b50 reperfusion.

We did not find interactions of various time courses with clinical outcome after 90 days, contradicting the results of the study of Kitano et al. [12]. They found decreasing association of good clinical outcome of mTICI 3 reperfusion over mTICI 2b after ≥80 min puncture to reperfusion time. It should be noted that our quartiles were different, with the upper quartile being >65 min puncture to reperfusion time, indicating differences between the two cohorts; however, with prolonged time courses, we found overall decreasing beneficial effects of successful reperfusion and also a decreasing likelihood of eTICI 2c/3 reperfusion. These findings make it reasonable to assume that longer onset to admission and/or longer procedure times promote tissue infarction and worse clinical outcomes as described before [18–21].

We found a trend of decreasing beneficial effect of complete recanalization in older patient groups, with the biggest leap being in the group with patients older than 82 years. This phenomenon is well known and probably relies on multiple factors, such as higher prevalence of comorbidities, higher rates of polypharmacy, and reduced neuronal plasticity [22–24]. Moreover, there is generally a less stringent association of successful reperfusion, initial neurological improvement and mid-term functional outcome in older patients [25].

Another interesting finding is that patients with either low NIHSS (≤9) or very high NIHSS (>19) did not benefit from eTICI 2c/3 as much as the group with NIHSS 10–19. A possible explanation for this could be that patients presenting with low NIHSS were more likely to have good to excellent collateral status and patients presenting with very high NIHSS were more likely to have poor collateral status. Previous studies showed the association of adequate collaterals and good tissue outcome [26, 27] and our results are in line with these findings, as the difference of eTICI 2b50/2b67 to eTICI 2c/3 reperfusion did not play a crucial role in patients with adequate collaterals. Also in favor of this hypothesis, patients in our cohort with NIHSS ≤5 benefited less from eTICI 2c/3 than patients with NIHSS 6–9. On the other hand, patients with poor collaterals benefited significantly more from a final eTICI 2c/3 than patients with good to excellent collaterals, underlining the importance of complete or near complete reperfusion especially in patients with poor collaterals.

While a beneficial effect of eTICI 2c/3 on the mRS at 90 days was present in ICA/T and M1 occlusions, it was absent in M2 occlusions. Obviously, eTICI 2b50/2b67 perfusion deficits after M2 occlusions are significantly smaller compared to the range of possible perfusion deficits in eTICI 2b50/2b67 reperfusion after e.g. M1 occlusions. Other possible explanations are different collateral situations in medium vessel occlusions and smaller amounts of affected brain tissue and therefore having a lower probability to affect more eloquent brain areas. In this sense, reliable measurement of the volume of remaining hypoperfused tissue during mechanical thrombectomy (e.g. with flat-panel CT perfusion) could help in deciding whether to extend or discontinue the procedure.

### Possible Implications on Patient Selection

Regarding extended mechanical thrombectomy for improving eTICI 2b50/2b67 to eTICI 2c/3, it is still not possible to draw clear boundaries for proper patient selection. In an individual case-based decision the operator may weigh in the likelihood of the improvement being clinically futile, which seems to occur more often in older patients, M2 occlusions and patients with either low or very high NIHSS.

### Limitations

The retrospective analysis of data from a single center implicates limitations of our study and generalizability of our results on other cohorts has to be validated. Furthermore, we found shorter time from symptom onset to groin puncture and lower number of retrieval attempts in patients with higher degrees of reperfusion, possibly biasing our findings. While we have corrected for these confounders in all presented logistic regression analyses, there was high collinearity between these variables and the possibility of residual and hidden confounding remains.

The results of our study should be interpreted with caution. It is not clear whether secondary established eTICI 2c/3 (after initial eTICI 2b50/2b67 reperfusion) over eTICI 2b50/2b67 has the same beneficial effects as first pass eTICI 2c/3, but recent reports seem promising [28, 29]. On the other hand, a lack of benefit of primary eTICI 2c/3 reperfusion over eTICI 2b50/2b67 indicates little to no benefit in secondary established eTICI 2c/3 reperfusion.

A particular strength of our study is the consistent core laboratory judication of the eTICI scores of our large sample size.

## Conclusion

The beneficial effect of eTICI 2c/3 over eTICI 2b50/2b67 was stable over various time metrics. It only decreased in older patient groups, M2 occlusions, patients with either low or very high NIHSS and patients with good to excellent collaterals. Improving eTICI 2b50/2b67 to eTICI 2c/3 in those subgroups may be more often futile, which should be acknowledged when making individualized decisions regarding potential improvements from eTICI 2b50/2b67 to eTICI 2c/3.

## Supplementary Information

Supplementary Table I
