# Supplementary material for: Heterogeneity of the Relative Benefits of TICI 2c/3 over TICI 2b50/2b67: Are there Patients who are less Likely to Benefit?
Source: Clin Neuroradiol. 2022 Jan 6;32(3):817–27. doi: 10.1007/s00062-021-01131-0 (PMC9424153; doi:10.1007/s00062-021-01131-0)
Supplement: Supplementary file 1 — Supplementary Table I [file 62_2021_1131_MOESM1_ESM.pdf]

Heterogeneity of the relative benefits of TICI 2c/3 over TICI 2b50/2b67: Are there Patients which are less likely to benefit?

ONLINE SUPPLEMENT

Supplementary Table I

	NIHSS ≤ 9 (n=235)	NIHSS 10-19 (n=447)	NIHSS ≥ 20 (n=221)	p
**ASITN/SIR DSA score (%)**				<0.001
**0**	3 (1.3)	27 (6.0)	31 (14.0)	
**1**	43 (18.3)	198 (44.3)	114 (51.6)	
**2**	61 (26.0)	135 (30.2)	54 (24.4)	
**3**	116 (49.4)	85 (19)	20 (9.0)	
**4**	12 (5.1)	2 (0.4)	2 (0.9)	

ASITN/SIR American Society of Intervention and Therapeutic Neuroradiology/Society of Interventional Radiology, *DSA* Digital subtraction angiography
